# Quantifying the distribution of protein oligomerization degree reflects cellular information capacity

**DOI:** 10.1038/s41598-020-74811-5

**Published:** 2020-10-19

**Authors:** Lena Danielli, Ximing Li, Tamir Tuller, Ramez Daniel

**Affiliations:** 1 Department of Biomedical Engineering, Technion-Israel Institute of Technology, 3200003 Haifa, Israel; 2 Department of Biomedical Engineering, Tel Aviv University, 69978 Ramat Aviv, Israel

**Keywords:** Computer modelling, Synthetic biology, Numerical simulations, Information theory

## Abstract

The generation of information, energy and biomass in living cells involves integrated processes that optimally evolve into complex and robust cellular networks. Protein homo-oligomerization, which is correlated with cooperativity in biology, is one means of scaling the complexity of protein networks. It can play critical roles in determining the sensitivity of genetic regulatory circuits and metabolic pathways. Therefore, understanding the roles of oligomerization may lead to new approaches of probing biological functions. Here, we analyzed the frequency of protein oligomerization degree in the cell proteome of nine different organisms, and then, we asked whether there are design trade-offs between protein oligomerization, information precision and energy costs of protein synthesis. Our results indicate that there is an upper limit for the degree of protein oligomerization, possibly because of the trade-off between cellular resource limitations and the information precision involved in biochemical reaction networks. These findings can explain the principles of cellular architecture design and provide a quantitative tool to scale synthetic biological systems.

## Introduction

A major goal of systems and computational biology is to gain understanding into the design principles underlying the complexity of large-scale biological networks at the organism level^1–4^ (e.g., metabolism, gene regulation, signal transduction, and protein–protein interaction). By performing computational analyses of statistical network properties, such as small-world^5^, scale-freeness^6^ and transitivity^7^ (global clustering coefficient), it has been shown that the connectivity of several protein–protein interaction (PPI) networks in living cells (e.g., *S. cerevisiae*^8^ and *H. pylori*^9^) and metabolic networks^10^ in various organisms (e.g., *A. fulgidus*,* E. coli* and *C. elegans*) have an inhomogeneous, scale-free network topology. Most proteins (i.e., nodes in networks) typically have few connections, and only some proteins have many connections with other proteins, and they are considered highly connected hubs. The degree distribution of these protein networks, defined by the probability of one protein to interact with other proteins, has been shown to follow a power law^8–10^.

Proteins in living organisms are often oligomers composed of multiple subunits, which may be identical (homo-oligomers) or different (hetero-oligomers). Homo-oligomers are prevalent in nature and play important roles in biology^11–14^. It has been shown that oligomerization can explain protein binding^14^, affecting the diversity and specificity of biochemical pathways^15,16^, and is associated with the regulation of enzyme activities^16,17^, cooperativity^18^ and stability^11^. Homo-oligomers often assume symmetric structures^11,19^, which allows proteins to form large structures without significantly increasing the genome size^20^.

From a biophysical perspective, cooperativity in living cells can be described as the number of identical or nearly identical components that collectively interact with each other to enhance and stabilize biochemical reactions^18^. Positive cooperativity increases with the increased affinity of ligands or protein binding and is modeled by the sigmoidal function ($$[x]^{n} /([x]^{n} + K_{d}$$), where [*x*] is the free ligand concentration, $$K_{d} $$ is the dissociation constant and *n* is known as the Hill coefficient^21^ (proportional to the degree of cooperativity). It has been shown that Hill coefficients and the sensitivity of biochemical reactions, defined as the ratio between the percentage of change in the input signal divided by the percentage of change in the output signal, are strongly correlated^18,22,23^. This correlation is widely used in the design of gene circuits by tuning their activities in response to external and internal signals. For example, transcription factors with multiple ligand-binding sites have more pronounced sigmoidal behaviors than transcription factors with a single binding site. While nature can utilize other biological mechanisms to increase the sensitivity of regulatory controls^24,25^, cooperativity is one means of scaling the complexity of cellular networks and improving their sensitivity^18^. However, these improvements in information quality are limited by the energy cost of protein synthesis^26^, and the balance between them can be achieved via cooperativity and protein oligomerization. For example, it has been shown that multiprotein complexes are created in proportion to stoichiometry, and the kinetics of individual proteins are optimized with regard to resource allocation and activity requirements. Similarly, it has recently been shown that a high transcription rate decreases stochastic fluctuations in gene expression but increases protein synthesis costs^27^.

In this work, we characterized the protein homo-oligomer frequencies in proteomes and observed that dimers are dominant, while the formation of larger oligomer decreases following a power law. This common oligomer distribution pattern was observed at the levels of both organisms and metabolic pathways and cannot be explained by free-scale network dynamics. Our results indicate that there is an upper limit for protein subunit number, possibly due to the trade-off between the energy cost of protein synthesis and the sensitivity of biochemical reactions. The delineation of the design principles underlying this distribution may reveal new insights into understanding cell functionality and its application in synthetic biology designs^28^.

## Results

### Protein homo-oligomer distribution

**Figure 1:**
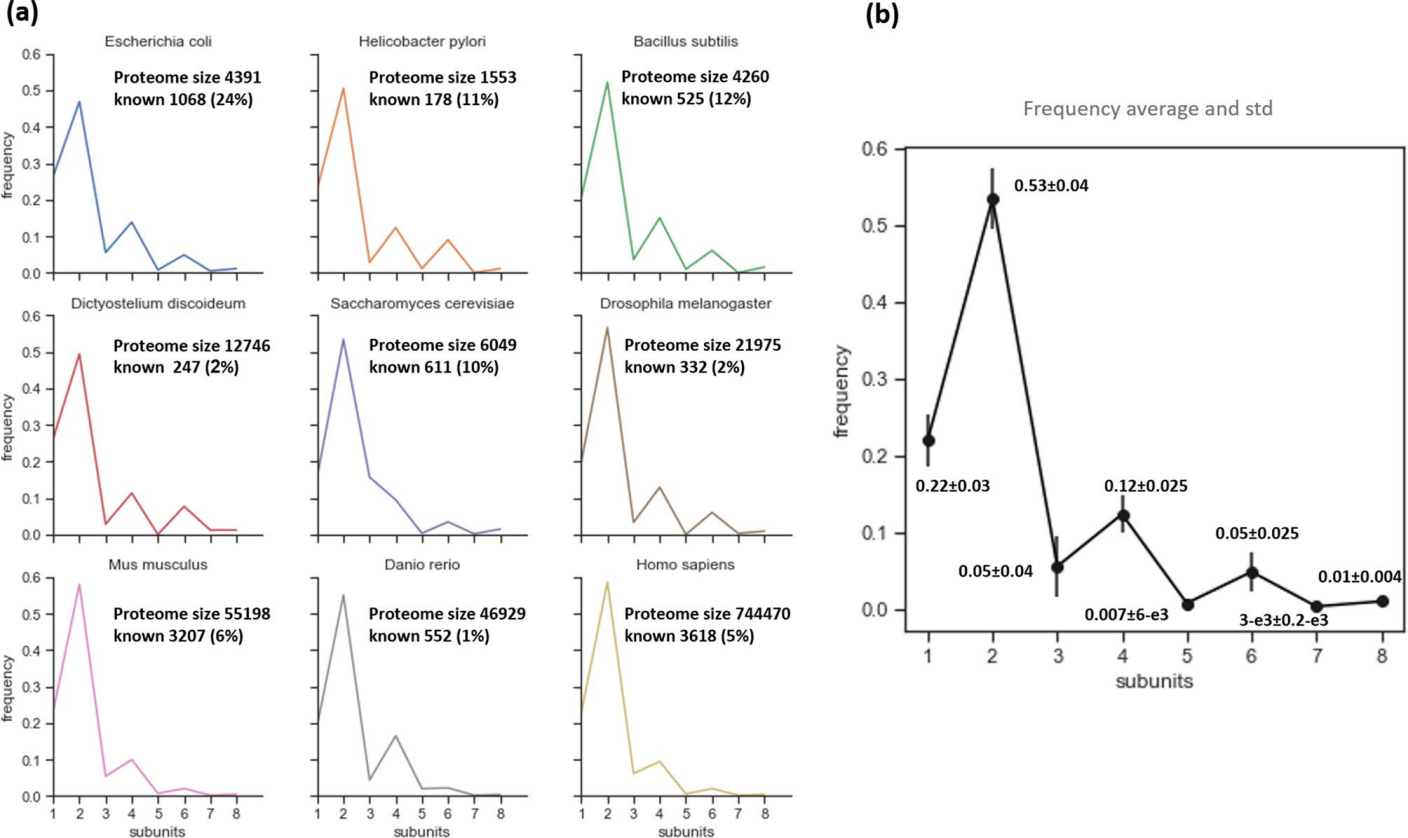
Homo-oligomer frequency in the proteome. (**a**) Homo-oligomer frequency in nine organisms: *E. coli*, *H. pylori*, *B. subtilis*, *D. discoideum*, *S. cerevisiae*, *D. melanogaster*, *M. musculus*, *D. rerio* and *H. sapiens*. The frequency was calculated based on the UniProt Knowledgebase proteomes. Proteome size and the number of known proteins with homo-oligomeric structure are listed for the nine organisms. (**b**) Average homo-oligomer frequency and standard deviation (std) for the nine organisms.

To explore the large-scale structure of protein oligomers, we characterized the homo-oligomer frequency of nine organisms (*E. coli*,* H. pylori*,* B. subtilis*,* D. discoideum*,* S. cerevisiae*,* D. melanogaster*,* M. musculus*,* D. rerio* and *H. sapiens)* based on UniProt Knowledgebase^29^ protein lists (Fig. 1a and Supplementary Fig. S1). The species tested in this study have been fully sequenced^30–34^, but only a small fraction of the oligomerization states is known. The presented results include both homo-oligomers, proteins assembled from an even number of identical subunits, and homo-oligomers, which are assembled with an odd number of units. Our analysis showed a common pattern of homo-oligomer frequency for the nine different species (Fig. 1b); the probability of observing proteins with *k* subunits in the proteome decreased as the value of *k* increased, and the number of odd subunits tend to be lower than the even subunits. Although a correlation between even and odd subunit composition and protein functionality was not established, it is known that the type of oligomer symmetry affects protein stability and functionality^11^. For example, cyclic symmetry is common for small oligomers (one or two subunits) with diverse functionality. The dihedral group comprises more stable proteins that have greater potential for interaction because of subunit interface diversity. Therefore, a possible explanation for fewer odd subunit compositions may be that compositions with an even number of subunits have the ability to form both cyclic and dihedral symmetries, while odd compositions tend to form only cyclic structures^35^. It is important to note the analogy of the subunit distribution with Oddo–Harkins rule^36,37^, which states that elements with even atomic numbers are more abundant in nature than elements with adjacently larger and smaller odd atomic numbers. It is assumed that protons in even numbers are paired to enhance nucleon stability by balancing each other’s spin, leading to even atomic number abundance. Moreover, element abundance decreases as the atomic number increases, showing similarity to the subunit number distribution.

### Protein homo-oligomer distribution for different categories of proteins based on Gene Ontology (GO)

To gain insights into subunit distributions for different protein classes, we calculated homo-oligomer frequencies based on GO categories. The GO system classifies gene products with respect to their molecular functions, cellular locations and biological processes^38^. In the current study, the subunit distribution was analyzed for *E. coli*, *B. subtilis*,* S. cerevisiae* and* M. musculus* (Supplementary Table S3 and Supplementary Fig. S2). Notably, GO annotations are related to genes (including all products of a gene) and not to proteins. Therefore, when a gene has different splice variants, the GO results are affected.

**Figure 2:**
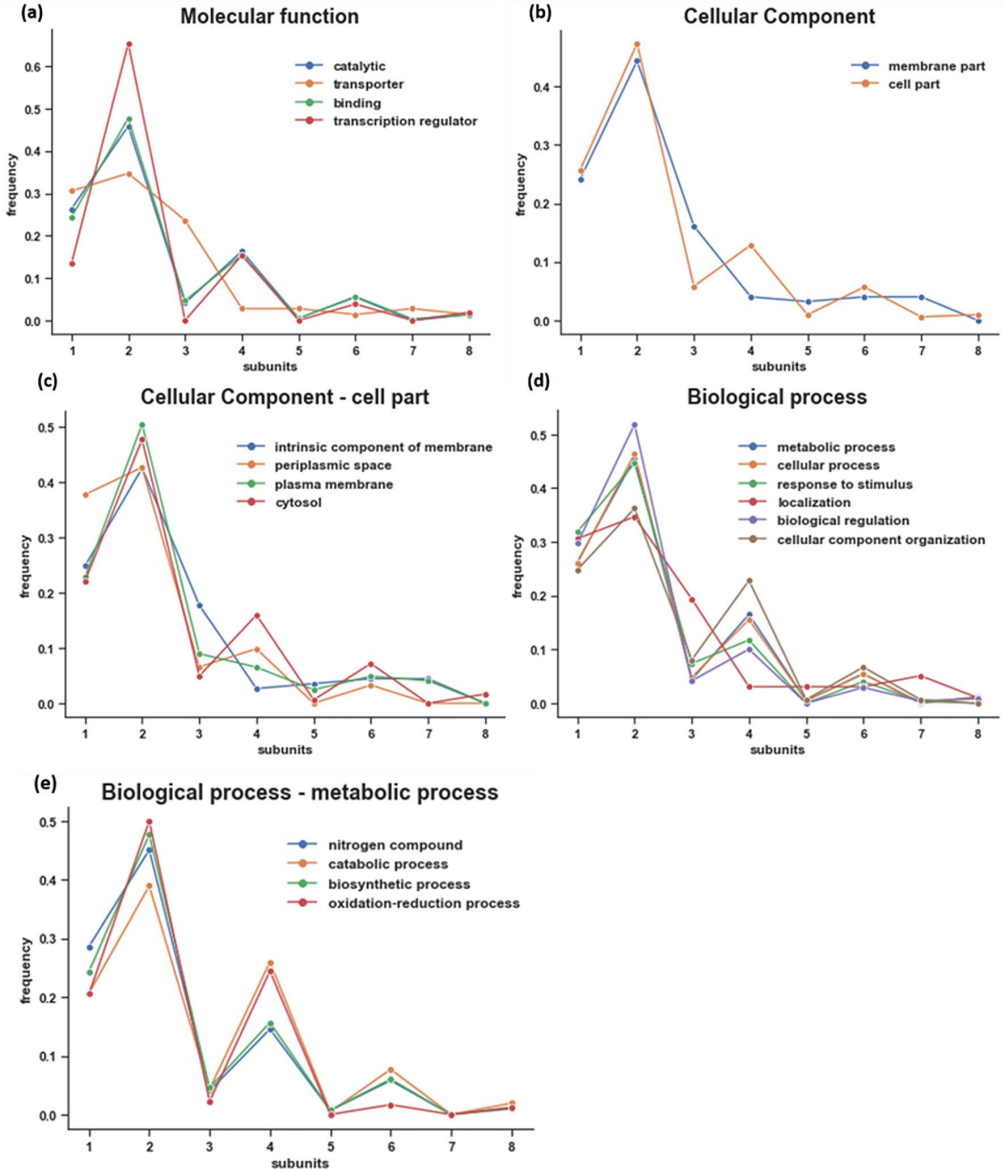
Protein subunit distribution for different GO classifications in *E. coli.* (**a**) Molecular function. (**b**) Cellular components such as membrane and cell compartment. (**c**) Different cell parts, such as intrinsic components of the membrane, periplasmic space, plasma membrane and cytosol. (**e**) Biological processes such as metabolic process, cellular process, response to stimulus, localization, biological regulation and cellular component organization. (**e**) Metabolic process, such as those involving nitrogen compounds, and catabolic, biosynthetic and oxidation–reduction processes. In addition, the protein subunit distribution for GO classes was calculated for *B. subtilis*,* S. cerevisiae* and *M. musculus* (see Supplementary Fig. S2). The conclusions are similar to those previously described for *E. coli*.

The homo-oligomer subunit distribution based on the protein classification for *E. coli* is shown in Fig. 2a. These proteins were classified into catalytic, transport, binding and transcription regulation categories. The protein homo-oligomer subunit distribution patterns, based on these protein categories, are similar to those found in the proteomes of the nine organisms studied herein (Fig. 1). The exception in *E. coli* is the set of transporter proteins, each of which is mostly composed of one, two or three subunits. Therefore, we suggest that, in *E. coli*, the choice of small subunit number was potentially preferred to enable the directed movements of substances within a cell or between cells and for rapid diffusion. The homogeneous distribution of tetramers and higher-order oligomers may be the result of functions that require larger structures, e.g., transport proteins that compose large passive bidirectional channels and aquaporins^11^. For example, the ammonia channel homodimer AmtB acts as an ammonium sensor and provides a sensitive mechanism by which ammonium flux into a cell is controlled^39,40^.

The subunit distributions in *E. coli* proteins classified by cellular location, such as membrane and cell compartments, are shown in Fig. 2b. The proteins located in the membrane and those located in cell compartments have similar patterns of monomer and homodimer distribution. However, for higher oligomers, the membrane protein distribution is similar to the distribution observed for transporter proteins because approximately 40% of membrane proteins are transporters.

Figure 2c shows the subunit distribution for the subclassifications of cell compartment: intrinsic membrane, periplasmic space, plasma membrane and cytosol. In these subclasses, we observed a similar lack of preference for even or odd subunit composition, except for proteins located in the cytosol; their subunit distribution follows the same pattern as the proteome.

The distribution of proteins classified by biological process, such as metabolic process, cellular process, response to stimulus, localization, biological regulation and cellular component organization is shown in Fig. 2d. In this case, the subunit distribution pattern was similar to that of the proteome with the exception of the localization process, because approximately 67% of proteins are transporters. All other metabolic processes (Fig. 2e), such as those involving nitrogen compounds, catabolic, biosynthetic and oxidation–reduction processes, exhibited distribution patterns typical of proteome subunit distribution. When we examined the subunit distribution according to protein classes, we found two types of patterns. One pattern was similar to the previously described distribution in the proteomes of the nine organisms. The second pattern was found in the subunit distribution of transporter proteins and proteins involved in localization processes, which did not show a preference based on even or odd subunits.

### Protein–protein interactions

**Figure 3:**
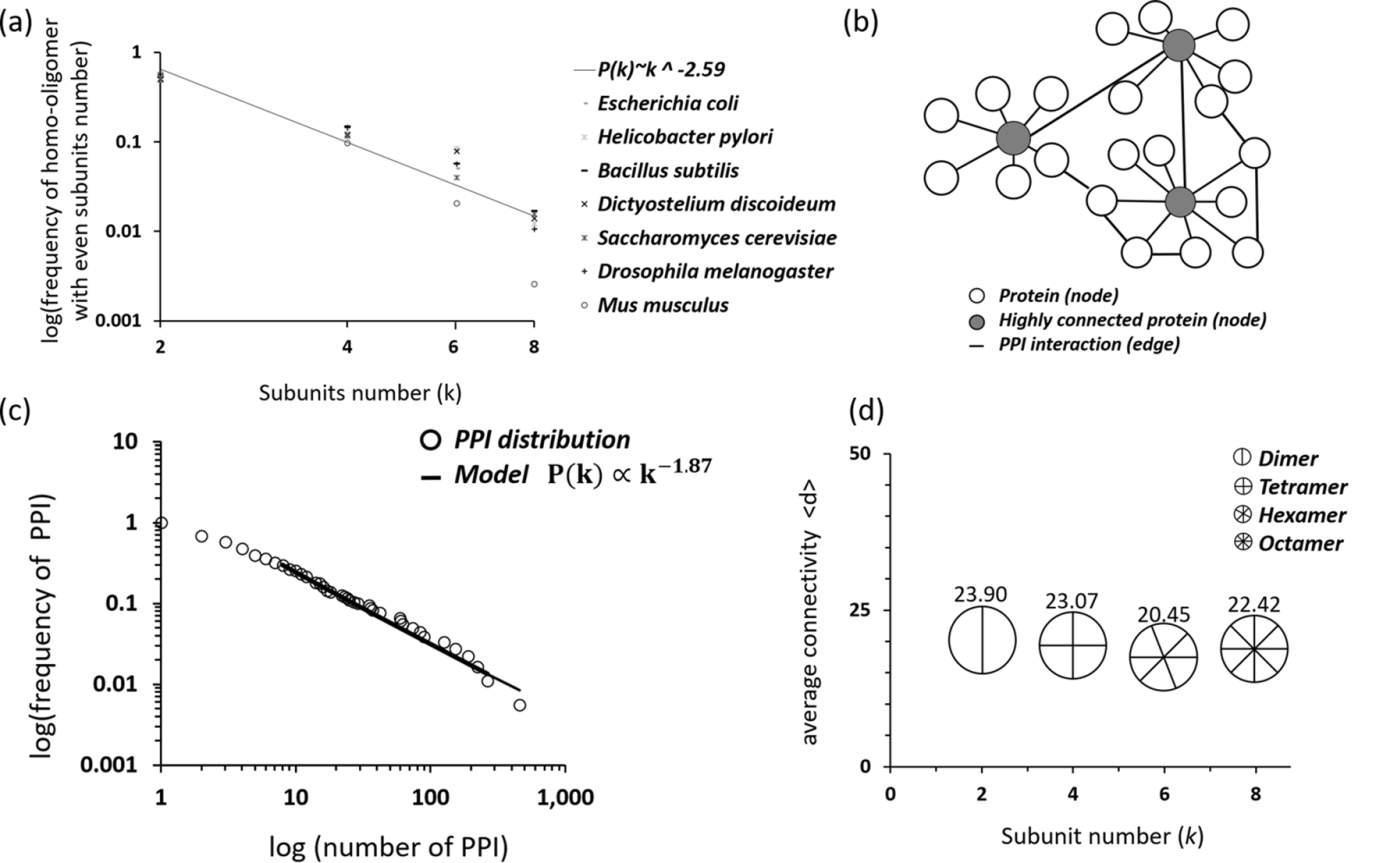
Protein–protein interactions. (**a**) The average probability of observing a protein with *k* subunits *P*(*k*) in homo-oligomers with an even number of subunits was fitted by a power law (*n* = 4, *P*(*k*) ~ *k*^−2*.*59^, *R*^2^ = 0.9, *p* = 0.003). (**b**) Graphic presentation of a free-scale power law network. The network is built of nodes, i.e., proteins, which are connected through undirected edges, which are functional interactions. White and gray circles represent proteins, and highly connected proteins are colored gray. Black lines represent interactions. (**c**) PPI networks followed a power law distribution of the form *P*(*k*) ∝ *k*^−1.87^ (*n* = 2875, *p* = 0.62, *k*_*min*_ = 8), where the *p* value corresponds to the Kolmogorov–Smirnov, *k*_min_ is the lower cut-off for the power law and *γ* = 1.87 (as expected, in the range 2 < *γ* < 3). (**d**) The average node degree <d> of even homo-oligomers with respect to protein subunit number.

The average probability of observing a protein with *k* subunits (*P*(*k*)) in homo-oligomers with an even number of subunits was fitted by the power law *P*(*k*) ~ − *k*^−2.59^ (*n* = 4, R^2^ = 0.93, p = 0.003) (Fig. 3a). Given that protein oligomerization determines the structural and functional aspects of proteins, we hypothesized that this distribution may be related to how subunits organize and interact in PPI networks. Furthermore, it was noted that the connectivity of a protein in a PPI network follows a power law distribution and is characteristic of scale-free networks. Following this observation, we first investigated whether the previously described distribution of protein oligomerization can be explained by the self-organization of large networks.

To confirm that protein connectivity in *E. coli* (K12 MG1655) PPI networks follows a power law, we generated an undirected graph of protein networks (Fig. 3b) using data from the STRING^41^ repository. The data included both experimental and predicted interactions, such as binding, inhibition, and activation reactions. In the graph presentation, the proteins are nodes which are connected to each other through undirected edges acting as functional interactions. As shown in Fig. 3c, the distribution of connectivity in the *E.coli* PPI network follows a power law. Consistent with the results in previous studies^8,9^, our results showed that the PPI was drawn based on a distribution in the form of $P\left( k \right) \propto k^{ - 1.87} \left( {n = 2875, p = 0.62, k_{min} = 8} \right)$, where the *p* value corresponds to the Kolmogorov–Smirnov test^42,43^, $k_{min}$ is the lower cut-off for the power law and *γ* = 1.87 (as expected, in the range *2* < *γ* < *3*). Here, the *p* value was used as a measure of the hypothesis suggesting that the power law is a good fit for the data, and *p* > 0.1 indicates that we cannot reject the hypothesis that the data were sampled from a distribution different than the power law. The network has an average node degree <*d*> = 23.3, where <*d*> is the average number of interactions of a protein in the network. In particular, <*d*> = 2*E⁄N*, where *E* is the total number of edges and *N* is the total number of nodes.

Initially, we attempted to explain the observed power law of oligomerization distribution (Fig. 2b) with the power law behavior of the PPI networks. If the distribution has a scale-free network property, then small oligomers (the majority) would be weakly connected nodes and large oligomers would be highly connected nodes (known as hubs). Therefore, we expected to see a correlation between protein subunit number and connectivity in the PPI networks. However, we found that the average connectivity of proteins, <*d*> , did not correlate with the subunit number. In contrast, the connectivity was homogenous across different oligomerization states (Fig. 3d). For example, the average degree of dimers (two subunits) was 23.9; for tetramers (four subunits), it was 23.07; for hexamers (sex subunits), it was 20.45; and for octamers (eight subunits), it was 22.42. While our results convincingly indicated the probability that the subunits of a specific number participate in *k* interactions following a power law distribution, the scale free distribution of the PPI networks failed to explain the power law pattern of the subunit distribution.

### Resource precision model

The development of systems with complex topology can be governed by two design principles, robustness and resource optimization. For example, it was suggested that networks that follow a scale-free power law distribution continue to operate properly even when several nodes are removed^6^. In addition, resource allocation in natural and synthetic networks limits a computation and strongly impacts information quality (precision). Thus, complex computational networks, such as those in the brain or electronics, are optimally evolved or designed based on trade-offs between resource consumption and precision^44^. The physical resources that a network utilizes for computation include space, time and energy efficiency per time unit (power) per part. These resources are related to each on the following basis^45^:

1 $$ func\left( {space} \right) \times func\left( {time} \right) \times func\left( {{\text{precision}}} \right) \propto power\,per\,part. $$

Equation (1) is known as a resource-precision model and is used to quantify resource consumption, when varying the precision of computed signals. The precision defined as the ratio between signal and system noise or effective number of resolution bits. The *func*(*time*) in Eq. (1) describes the speed or the time required to compute the task, and *func*(*space*) is proportional to the number of devices, nodes and parts needed to perform the computation. The frequency of a protein with *k* subunits represents the periodical appearance of the *k*-subunit structure across the proteome (space); therefore, the frequency can be interpreted as the spatial frequency of the k-subunit structure (Fig. 1b). This relationship holds true for the frequency of a protein with *k* subunits across the total number of copies of the whole proteins in the cell (protein abundance) (Supplementary Fig. S6). For this reason, a simple form of Eq. (1) assumes that proteins with *k* subunit frequencies explain the *func*(*space*) and reflect the relative number of parts in the calculations performed in a certain cell (space) as follows:

2 $$ f_{s} \times f \times func\left( {{\text{precision}}} \right) \propto power\,per\,part, $$

where *f*_*s*_ is the fraction (empirical probability) of a certain part in a specific space (the ratio between the number of specific parts *N* and total number of parts *N*_*T*_ in the entire computing system), and *f* is the frequency in time (the ratio between the number of specific events and the total number of events).

From an evolutionary perspective, specifically from the point of view of unicellular processes in multicellular organisms, time is a continuous and infinite resource for life expression. Thus, for simplicity, we assume that the time is not a degree of freedom and is not included in the resource of energy allocation, and thus, we write:

3 $$ f_{s} \times func\left( {{\text{precision}}} \right) \propto energy\,per\,part. $$

To explain the power law distribution of protein oligomerization, we fit the resource-precision model (Eq. 3) to molecular and genetic networks in living cells. We first calculated the energy cost of protein (with identical *k* subunits) synthesis in living cells, for which typically as much as one-third of the total energy is produced during cell replication^46^. Moreover, there are estimates suggesting that translation consumes more than 70% of cell energy^47^. The energy cost *E*(*k*) was calculated assuming the consumption of 4.5 ATP molecules per amino acid (aa)^48,49^ and the average length of a protein, L, to be ~ 360 aa in eukaryotes and ~ 270 aa^50^ in bacteria, as follows:

4 $$ E\left( k \right) \propto 4.5 \times L \times k . $$

**Figure 4:**
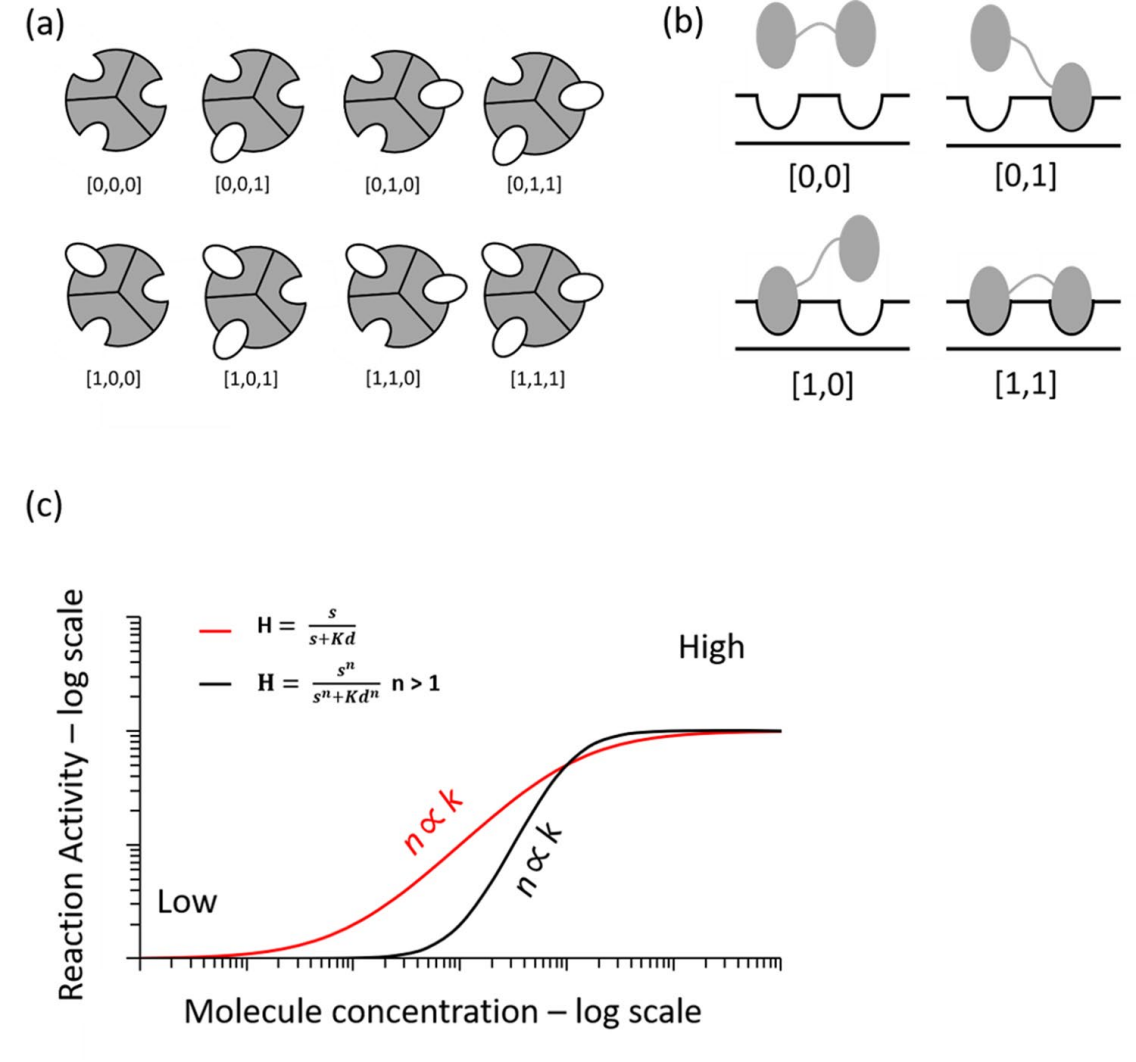
Biochemical binding reactions. (**a**) Schematic presentation of an enzyme that contains three identical subunits, and each subunit contains a ligand-binding site. The reaction between a small molecule and the enzyme can be represented by 8 (2^3^) statistical arrangements. (**b**) Schematic presentation of a DNA-binding dimer. The binding reaction can be represented by 4 (2^2^) statistical arrangements. (**c**) The precision of the biochemical reaction. An ultrasensitive response indicates that a small change in stimulus causes a large change in response and produces a sigmoidal dose–response curve (black line). An ultrasensitive response is described by the Hill equation when the Hill coefficient is *n* > 1. The red line represents the Michaelis–Menten equation when the Hill coefficient is *n* = 1. The Hill equation *H* = *s*^*n*^*/*(*s*^*n*^ + *Kd*), where *s* is the unbound protein concentration, *Kd* is the dissociation constant and *n* is the coefficient that measures “ultrasensitivity” or cooperativity of the biochemical reactions. The black curve represents a reaction that requires a lower molecule concentration, from low to high, than is indicated by the red curve to activate the biochemical reaction.

Proteins interact with various cellular components, such as DNA, RNA, proteins and small molecules, to produce specific cellular responses. These interactions often involve sets of biochemical reactions, which integrate continuous biological signals and discrete states [ON, OFF]. Figure 4a depicts an enzyme generated from three identical subunits, each of which bears a binding site for a small molecule (ligand). The biochemical reactions between the small molecule and the enzyme are represented by as many as eight statistical arrangements. Figure 4b depicts a DNA-interacting dimer, in which either one subunit binds to DNA or both subunits bind to DNA. The binding reaction can be represented by four statistical arrangements. This assumption suggests that if one or more subunit binding sites are occupied, then the state is 1; otherwise, the state is 0. Ideally, we should assume the existence of more than two states and consider the intermediate levels between the states. Therefore, in this case, the biochemical reactions between the small molecule and the enzyme can be represented by M^k^ (M > 2) statistical arrangements. However, for simplicity, we selected 2^k^ states based on the best empirical fit of our model (Supplementary Fig. S4). Accordingly, the precision of the biochemical reaction is expected to be proportional to the number of possible states:

5 $$ Prec \propto \frac{1}{{2^{k} }}. $$

**Figure 5:**
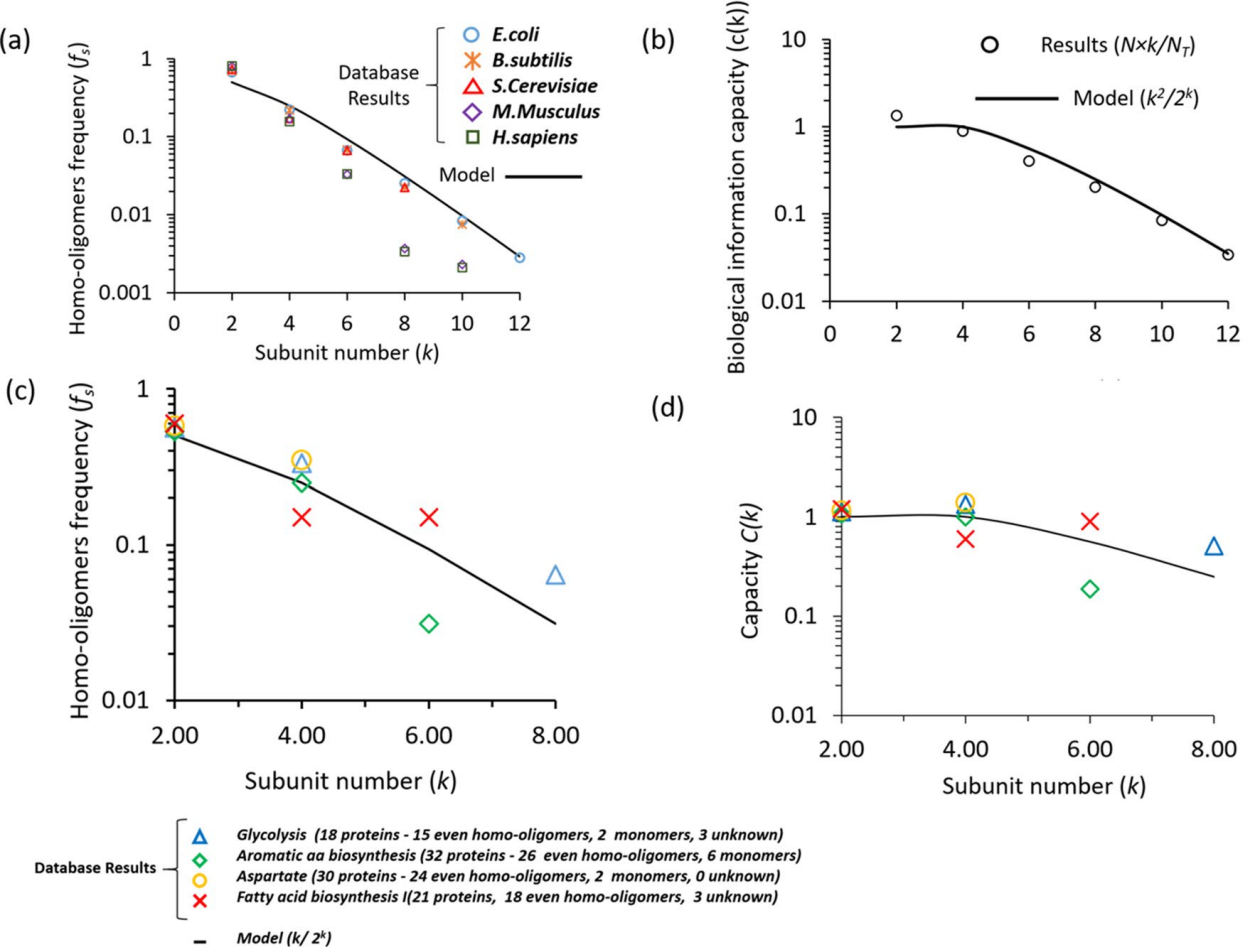
Homo-oligomer distribution model. (**a**) Fitting of protein with an even number of subunits to a homo-oligomer distribution (*f*_*s*_ = *N/N*_*T*_) using the resource-precision model *(f*_*s*_* ∝ k/*2^*k*^), where *N* is the number of proteins with *k* identical subunits and *N*_*T*_ is the total number of proteins found in an organism. (**b**) Fitting of the biological information capacity (*c*(*k*)) by the capacity model *c*(*k*) = *f*_*s*_ × *k*, where fs is the homo-oligomer distribution (*f*_*s*_ = *N/N*_*T*_) and *k* = *log*_2_(*M*) is the bit number (*M* = 2^*k*^ is the signal level). (**c**) Fitting of the protein with an even number of subunits to a homo-oligomer distribution (*f*_*s*_ = *N/N*_*T*_) in biological pathways, as determined by resource precision. Several monomers and proteins involved in these pathways but with unknown degrees of oligomerization were neglected. (**d**) Biological information capacity of the pathways.

Equation (5) shows that when the number of subunits increases, the precision level of a biochemical reaction decreases, resulting in a higher degree of exactness. Thus, a system with high precision can detect weak signals, which implies that proteins with more subunits are capable of detecting small changes in inputs (i.e., ultrasensitivity^23^, Fig. 4c). On the other hand, when the number of subunits increases, the energy cost increases according to Eq. (4). Thus, we expect a trade-off between precision and energy cost. Substituting the precision term (Eq. 5) and the energy term (Eq. 4) into the resource-precision model (Eq. 3), we find that the frequency appearance of protein homo-oligomers (*f*_*s*_ = *N/N*_*T*_) in living cells is given by:

6 $$ f_{s} \propto \frac{k}{{2^{k} }}, $$

where *N* is the number of proteins with *k* identical subunits and *N*_*T*_ is the total number of proteins found in an organism. As in Fig. 5a, our model (Eq. 6) fits well with the database results of the tested organisms (Supplementary Fig. S4). Additionally, the frequency of protein homo-oligomers was estimated from the protein abundance level (typical number of proteins in the cell) for eight different organisms and found to follow a similar pattern as frequency over the proteome (Supplementary Fig. S6). Furthermore, the product *c*(*k*) = *f*_*s*_(*k*) × *k* is proportional to the total number of parts that appear in the proteome with *k* subunits (Fig. 5b).

In information theory, *k* = *log*_2_(*M*) is equivalent to the number of bits, and *M* = *2*^*k*^ is the signal level number, where *f*_*s*_ is the bandwidth, which is defined as the difference between the upper and lower bounds for signal frequencies. Thus, the product *c*(*k*) = *f*_*s*_(*k*) × *k* is analogous to information capacity, which explains the trade-off between precision and energy cost. It is also known that the Nyquist information theory of a noise-free channel^51^ sets a limit on the maximum rate at which information can be transmitted over a communication channel. Such rate limitations arise from a trade-off between computation and communication. Thus, the maximum signal level that the receiver can distinguish is limited by the capacity of a communication channel. Analogously, in biological networks, a DNA promoter or any other protein involved in reactions can act as the receiver. The activity of a promoter containing several binding sites would thus place a substantial metabolic burden on a cell compared to a promoter with a single binding site.

To gain deeper insights into the conditions of the resource–precision model, we further explored four metabolic pathways in living cells: glycolysis and aspartate, aromatic amino acid and fatty acid biosynthesis. We analyzed the even homo-oligomerization degree of the above metabolic pathways based on the EcoCyc^52–54^ *E. coli* data set. The protein oligomerization distributions in these essential metabolic pathways also followed a similar distribution pattern (Fig. 5c), consistent with the distribution of the organism’s proteome. Furthermore, the information capacity *c*(*k*) of these pathways was well matched by the model of biological information capacity (Fig. 5d, Eq. 6).

Remarkably, in our model, we approximated the total number of statistical arrangements for a protein with *k* subunits as 2^*k*^ arrangements, which is an upper bound value. An alternative model of self-assembling cyclic protein homo-oligomers^55^ can describe the total arrangements using the Necklace function, i.e.,$\frac{1}{k}\sum\nolimits_{i = 1}^{i = v\left( k \right)} \varphi \left( {d_{i} } \right)2^{{k/d_{i} }}$, where *d*_*i*_ are the divisors of *k* and $\varphi \left( {d_{i} } \right)$ is the Euler totient function^56^. Therefore, the $2^{k}$ term in Eq. (6), which also appears in the Necklace function, is sufficient to describe the statistical arrangements.

## Discussion

In this work, we developed a resource–precision model (Eq. 6) that predicts protein homo-oligomerization distribution in living cells. The biological information, which is represented by the combinatorial discrete (logic) levels of protein subunits, sets the computational precision of biochemical reactions (e.g., protein–protein interactions, protein–DNA interactions, and protein–small molecule interactions). The model indicates that there is a trade-off between information quality, energy cost of protein synthesis and resource allocation. For example, reactions that maximize information precision might require a high degree of homo-oligomerization, which places a substantial metabolic burden on a cell. Proteins involved in these reactions are rare in the proteome. The proposed resource–precision model was adopted in other established engineering disciplines to solve challenges of design trade-offs^57^.

However, not all protein signals follow observable patterns. For example, homo-oligomer distribution over several pathways in *S. cerevisiae* (Supplementary Fig. S5) or high relative abundance of homodimers in *E. coli* (Supplementary Fig. S6) cannot be explained by a resource precision model. In future research, we need to explore metabolic pathways for more prokaryotic and eukaryotic organisms to try and better understand which types of pathways match our model. In addition, the whole functional group of metabolic pathways (e.g., metabolism of nucleotides) and connected pathways [e.g., glycolysis, citrate cycle (TCA cycle), and pyruvate metabolism] can be studied for a deeper understanding of biological functionality. Moreover, the effects of unusual constraints on evolution, weak evolutionary selection (e.g., due to small effective population sizes) or a nonequilibrium state of the genome (e.g., after whole-genome duplication) can be explored as possible explanations for the genomes for which our suggested model is less relevant.

The common rules underlying protein oligomerization distribution can help better understand biological processes in nature and reveal new design principles of cellular architecture. The challenging tasks of synthetic biology include scale-up protein network generation and robust computation performance for living cells for use in diagnostic, therapeutic and biotechnological applications^58–60^. Expanding our knowledge of protein oligomerization in the context of single molecules, multimolecular networks and whole cells will contribute to new levels of understanding of the critical roles that cooperativity play in the function of complex biological systems.

Developing an advanced model that takes into consideration the diffusion, channel noise and analog signals of proteins can be included in future works (e.g., Shannon information theory^51,61^). Based on this model, we can design complex genetic circuits and compare their performances with common state-of-the art designs. This experiment can also help to find ways to reduce the component number in design and improve efficiency by combining circle parts (proteins) with respect to the frequency pattern observed in the proteome of some organisms.

## Methods

### Calculating the distribution of protein homo-oligomers in the proteome

Protein homo-oligomer distribution was calculated based on UniProt proteomes^29^. When the protein oligomerization state was known, it was found in the protein interaction section of the subunit structure subsection [CC]. For this experiment, we used the full proteome of nine organisms (Supplementary Table S2). Protein homo-oligomers were grouped by subunit number (Supplementary Table S1), and then, the protein fraction of each group was calculated from the total numbers of known homo-oligomers. All data and detailed calculations can be found and reproduced from the open source Git-Hub repository (Supplementary Methods S1).

### Calculating the distribution of protein homo-oligomers in the proteome based on GO classification

Protein homo-oligomer distribution for different categories of proteins based on GO^38,62^ was calculated based on UniProt proteome data^29^. For this experiment, we used the full proteome of four organisms (Supplementary Fig. S2). Protein homo-oligomers were grouped into GO categories and then subgrouped by subunit number. The protein fraction of each GO class and each subunit subgroup was calculated from the total number of known homo-oligomers in the GO category. All data and detailed calculations can be found and reproduced from the open source Git-Hub repository (Supplementary Methods S1).

### Calculating the distribution of protein–protein interactions for *E. coli*

The protein–protein interaction probability distribution for *E. coli* was calculated based on data from the STRING^41^ repository file, which includes the following columns: ecoli_interaction_id_a, ecoli_interaction_id_b, ecoli_interaction_mode, ecoli_interaction_action, a_is_acting, score. The columns ecoli_interaction_id_a and ecoli_interaction_id_b contain interacting protein names. The unique ID list and counts per ID were calculated using the data in column ecoli_interaction_id_a. Then, the connection probability for each protein ID was calculated using the total connection number (p(k) = protein connection\total connections). The calculation of the connection probability function for each subunit number k was calculated in the same way, but the proteins were categorized based on homodimer, homotetramer, homohexamer and homo-octamer groups. The data file 511145.protein.actions.v10.xlsx can be found in a protein-homo-oligomer-distribution repository (Supplementary Transparence Methods) in the *E. coli* folder.

## Supplementary information

Supplementary Information.

## Data Availability

The data sets analyzed in the current study are available in the protein-homo-oligomer-distribution repository, https://github.com/LenaDanielli/Protein-homo-oligomer-distribution.
