# Author Correction: Quantifying the distribution of protein oligomerization degree reflects cellular information capacity

**DOI:** 10.1038/s41598-021-86894-9

**Published:** 2021-03-26

**Authors:** Lena Danielli, Ximing Li, Tamir Tuller, Ramez Daniel

**Affiliations:** 1 Department of Biomedical Engineering, Technion-Israel Institute of Technology, 3200003 Haifa, Israel; 2 Department of Biomedical Engineering, Tel Aviv University, 69978 Ramat Aviv, Israel

Correction to: *Scientific Reports* https://doi.org/10.1038/s41598-020-74811-5, published online 19 October 2020

The original version of this Article contained a typographical error in the spelling of the author Tamir Tuller, which was incorrectly given as Tamir Tuler. This has now been corrected in the PDF and HTML versions of the Article, and in the accompanying Supplementary Information file.

