# Supplementary material for: Quantifying the distribution of protein oligomerization degree reflects cellular information capacity
Source: Sci Rep. 2020 Oct 19;10:17689. doi: 10.1038/s41598-020-74811-5 (PMC7573690; doi:10.1038/s41598-020-74811-5)
Supplement: Supplementary file 1 — Supplementary Information. [file 41598_2020_74811_MOESM1_ESM.docx]

**Supplemental Information**

Quantifying the distribution of protein oligomerization degree reflects cellular information capacity

Lena Danielli^1^, Ximing Li^1^, Tamir Tuller^2^ and Ramez Daniel^1*^

^1^Department of Biomedical Engineering, Technion – Israel Institute of Technology, Haifa, 3200003, Israel

^2^Department of Biomedical Engineering, Tel Aviv University, Ramat Aviv, 69978, Israel

***Corresponding author**

Email: [ramizda@bm.technion.ac.il](mailto:ramizda@bm.technion.ac.il); Phone: 972-502129693

# Tables

## Supplementary Table S1: Protein homo-oligomers nomenclature

| Formal name | Short name | Number of subunits |
| --- | --- | --- |
| Monomer | 1-mer | 1 |
| Homodimer | 2-mer | 2 |
| Homotrimer | 3-mer | 3 |
| Homotetramer | 4-mer | 4 |
| Homopentamer | 5-mer | 5 |
| Homohexamer | 6-mer | 6 |
| Homoheptamer | 7-mer | 7 |
| Homooctamer | 8-mer | 8 |

## Supplementary Table S2: Organisms proteomes repository

A proteome is the set of proteins expressed by an organism. Proteomes data was downloaded from freely accessible UniProt repository^1^.

| Name | ID | Type | Link |
| --- | --- | --- | --- |
| *E. coli* | up000000625 | Prokaryote | <https://www.uniprot.org/proteomes/UP000000625> |
| *H. pylori* | up000000429 | Prokaryote | https://www.uniprot.org/proteomes/UP000000625 |
| *B. subtilis* | up000001570 | Prokaryote | [https://www.uniprot.org/proteomes/UP000001570](https://www.uniprot.org/proteomes/UP000000625) |
| *D. discoideum* | up000002195 | Eukaryote | [https://www.uniprot.org/proteomes/UP000002195](https://www.uniprot.org/proteomes/UP000000625) |
| *S. cerevisiae* | up000002311 | Eukaryote | [https://www.uniprot.org/proteomes/UP000002311](https://www.uniprot.org/proteomes/UP000000625) |
| *D. melanogaster* | up000000803 | Eukaryote | [https://www.uniprot.org/proteomes/UP000000803](https://www.uniprot.org/proteomes/UP000000625) |
| *M. musculus* | up000000589 | Eukaryote | [https://www.uniprot.org/proteomes/UP000000589](https://www.uniprot.org/proteomes/UP000000625) |
| *D. rerio* | up000000437 | Eukaryote | [https://www.uniprot.org/proteomes/UP000000437](https://www.uniprot.org/proteomes/UP000000625) |
| *H. sapiens* | up000005640 | Eukaryote | [https://www.uniprot.org/proteomes/UP000005640](https://www.uniprot.org/proteomes/UP000000625) |

## Supplementary Table S3: Gene Ontology (GO)

GO^2,3^ classify proteins with respect to their molecular function, cellular location/component and biological process. *E. coli* (up000000625), *B. subtilis* (up000001570), *S cerevisiae* (up000002311), *M. musculus* (up000000589), proteome was classified based on GO.

| Class | GO no. | Sub classes | GO no. | Sub classes | GO no. |
| --- | --- | --- | --- | --- | --- |
| Molecular function | GO:0003674 | catalytic | GO:0003824 |  |  |
|  |  | transporter | GO:0005215 |  |  |
|  |  | binding | GO:0005488 |  |  |
|  |  | transcription regulator | GO:0140110 |  |  |
| Cellular Component | GO:0005575 | membrane part | GO:0044425 |  |  |
|  |  | cell part | GO:0044464 | intrinsic component of membrane | GO:0031224 |
|  |  |  |  | periplasmic space | GO:0042597 |
|  |  |  |  | plasma membrane | GO:0005886 |
|  |  |  |  | cytosol | GO:0005829 |
| Biological process | GO:0008150 | cellular component organization | GO:0071840 |  |  |
|  |  | response to stimulus | GO:0050896 |  |  |
|  |  | localization | GO:0051179 |  |  |
|  |  | biological regulation | GO:0065007 |  |  |
|  |  | metabolic process | GO:0008152 | nitrogen compound | GO:0006807 |
|  |  |  |  | catabolic process | GO:0009056 |
|  |  |  |  | biosynthetic process | GO:0009058 |
|  |  |  |  | oxidation-reduction process | GO:0055114 |

## Supplementary Table S4: Organisms proteomes repository for protein abundance calculation

A proteome is the set of proteins expressed by an organism. Proteomes data was downloaded from Protein Abundance Database PaxDb^4^.

| Name | ID | Coverage | Score | File |
| --- | --- | --- | --- | --- |
| *E. coli* | 137 | 99 | 23.08 | 511145-WHOLE_ORGANISM-integrated.txt |
| *M. tuberculosis* | 253 | 85 | 12.33 | 83332-WHOLE_ORGANISM-integrated.txt |
| *B. henselae* | 437 | 86 | 9.2 | 283166-Bhenselae_Albrethsen_2013.txt |
| *S. pombe* | 250 | 90 | 18.49 | 4896-WHOLE_ORGANISM-integrated.txt |
| *S. cerevisiae* | 3 | 96 | 21.24 | 4932-WHOLE_ORGANISM-integrated.txt |
| *R. norvegicus* | 93 | 73 | 14.59 | 10116-WHOLE_ORGANISM-integrated.txt |
| *M. musculus* | 196 | 89 | 14.15 | 10090-WHOLE_ORGANISM-integrated.txt |
| *H. sapiens* | 29 | 87 | 15.5 | 9606-WHOLE_ORGANISM-integrated.txt |

## Supplementary Table S5: Organisms proteomes repository for protein abundance mapping

A proteome is the set of proteins expressed by an organism. Proteomes data was downloaded from freely accessible UniProt repository^1^.

| Name | ID | Type | Link |
| --- | --- | --- | --- |
| *E. coli* | up000000625 | Prokaryote | <https://www.uniprot.org/proteomes/UP000000625> |
| *M. tuberculosis* | up000001584 | Prokaryote | https://www.uniprot.org/proteomes/UP000001584 |
| *B. henselae* | up000058422 | Prokaryote | <https://www.uniprot.org/proteomes/UP000058422> |
| *S. pombe* | up000002485 | Eukaryote | <https://www.uniprot.org/proteomes/UP000002485> |
| *S. cerevisiae* | up000002311 | Eukaryote | [https://www.uniprot.org/proteomes/UP000002311](https://www.uniprot.org/proteomes/UP000000625) |
| *R. norvegicus* | up000002494 | Eukaryote | [https://www.uniprot.org/proteomes/UP000000803](https://www.uniprot.org/proteomes/UP000000625) |
| *M. musculus* | up000000589 | Eukaryote | [https://www.uniprot.org/proteomes/UP000000589](https://www.uniprot.org/proteomes/UP000000625) |
| *H. sapiens* | up000005640 | Eukaryote | [https://www.uniprot.org/proteomes/UP000005640](https://www.uniprot.org/proteomes/UP000000625) |

# Figures

## Supplementary Figure S1: Homo-oligomer frequency

Homo-oligomer frequency in nine organisms: *E. coli, H. pylori, B. subtilis, D. discoideum, S. cerevisiae, D. melanogaster, M. musculus, D. rerio (Zebrafish)* and *H. sapiens*. The frequency was calculated based on the UniProt^1^ knowledgebase proteome.


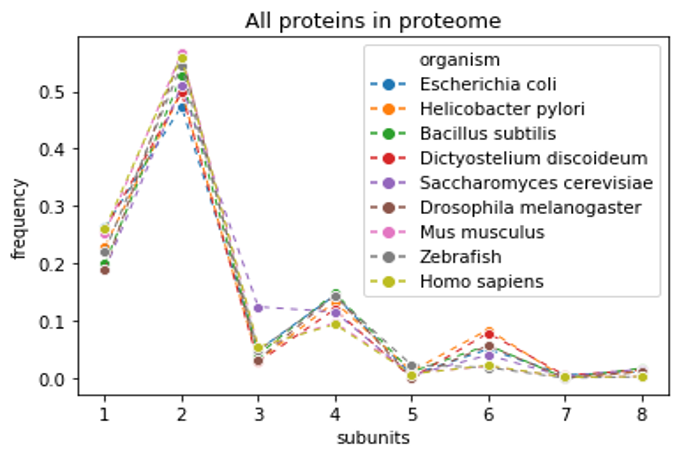


## Supplementary Figure S2: Gene Ontology (GO)

Protein oligomers frequency for GO^2,3^ classes and subclasses of proteins with respect to their molecular function, cellular location/component and biological process. *E.coli* (up000000625), *B. subtilis* (up000001570), *S. cerevisiae* (up000002311), *M. musculus* (up000000589), proteome was classified based on GO. X axis represents oligomer frequency and Y axis represents subunits number. Each column related to one organism and each row to specific GO class.


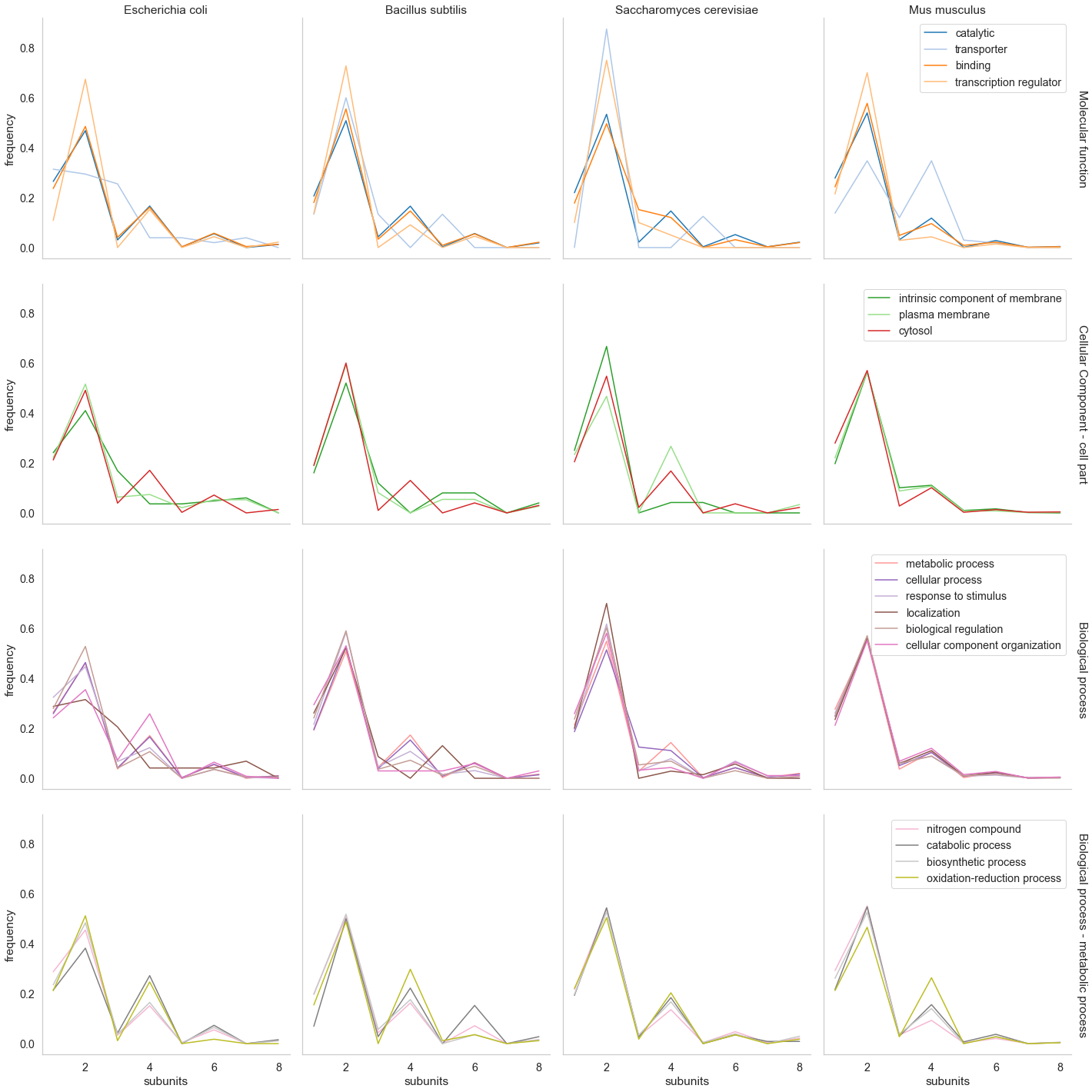


## Supplementary Figure S3: Protein-protein interactions

Protein-protein interactions (PPI) distribution in *E. coli* for 4 homo-oligomeric protein states – dimeric, tetrameric, hexameric and octameric. From these results we can see that protein homo-oligomers connectivity is not related to their oligomeric state. There are differences in distribution among the groups that can be result of various proteins functional needs. In each group there are proteins with only few connections and also highly connected ones that have about 100 connections.


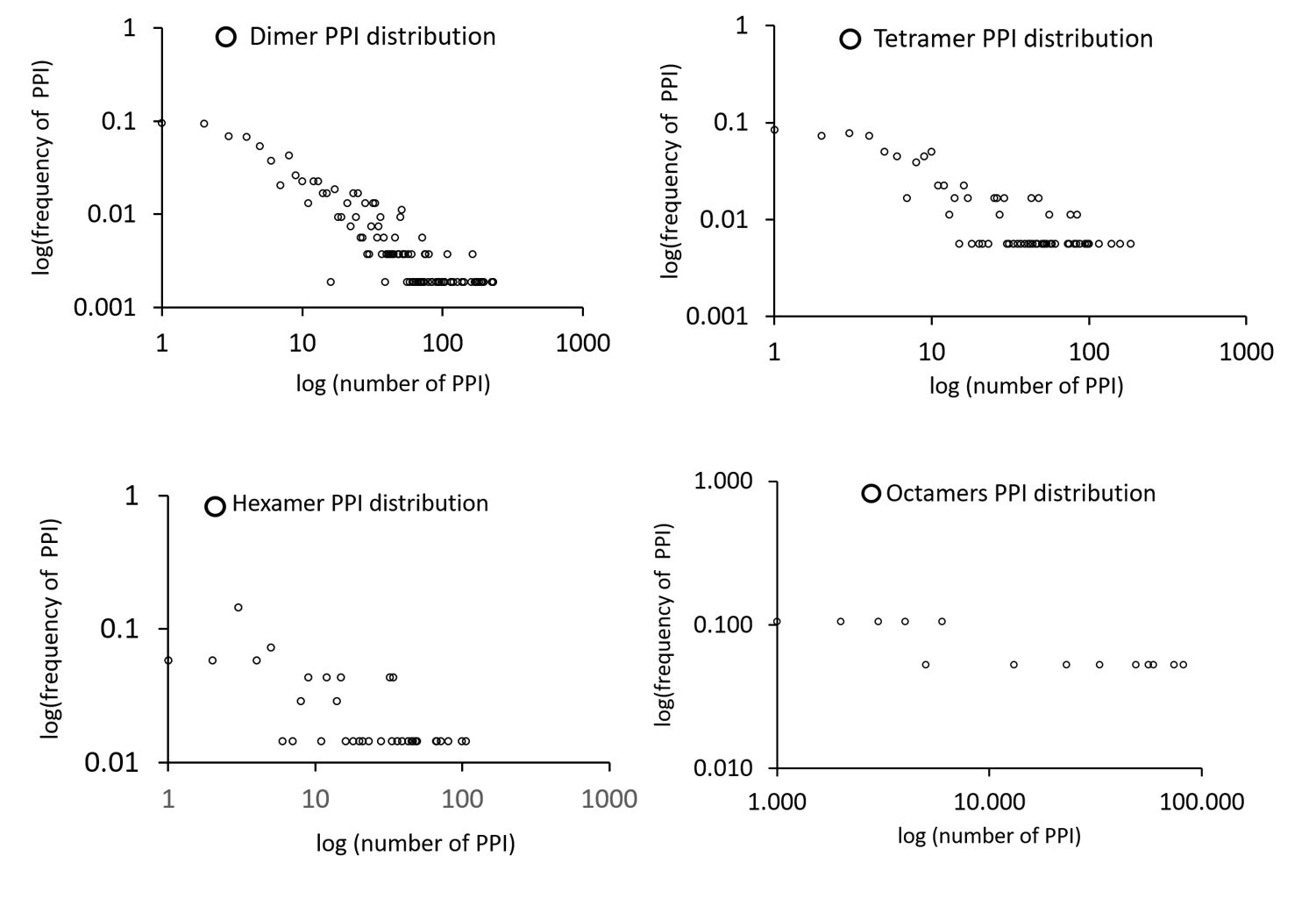


## Supplementary Figure S4: Resources precision model fitting

(a) Protein with k subunits average frequency was fitted using resources precision model *f(k)* = *k/x^k^*, when *x* = 2, 3, 4 and 5. (b) Model power was evaluated by coefficient of determination (*R*^2^) and significance (*p-value*). The minimal mean absolute fitting error (*MAE*) was used to choose best model. All models give significant results (*R*^2^ > 0.9, *p* < 0.05), but *x* = 2 give minimal *MAE* = 0.05. Based on this finding we selected *f(k)* = *k/2^k^* as best model to fit our data.


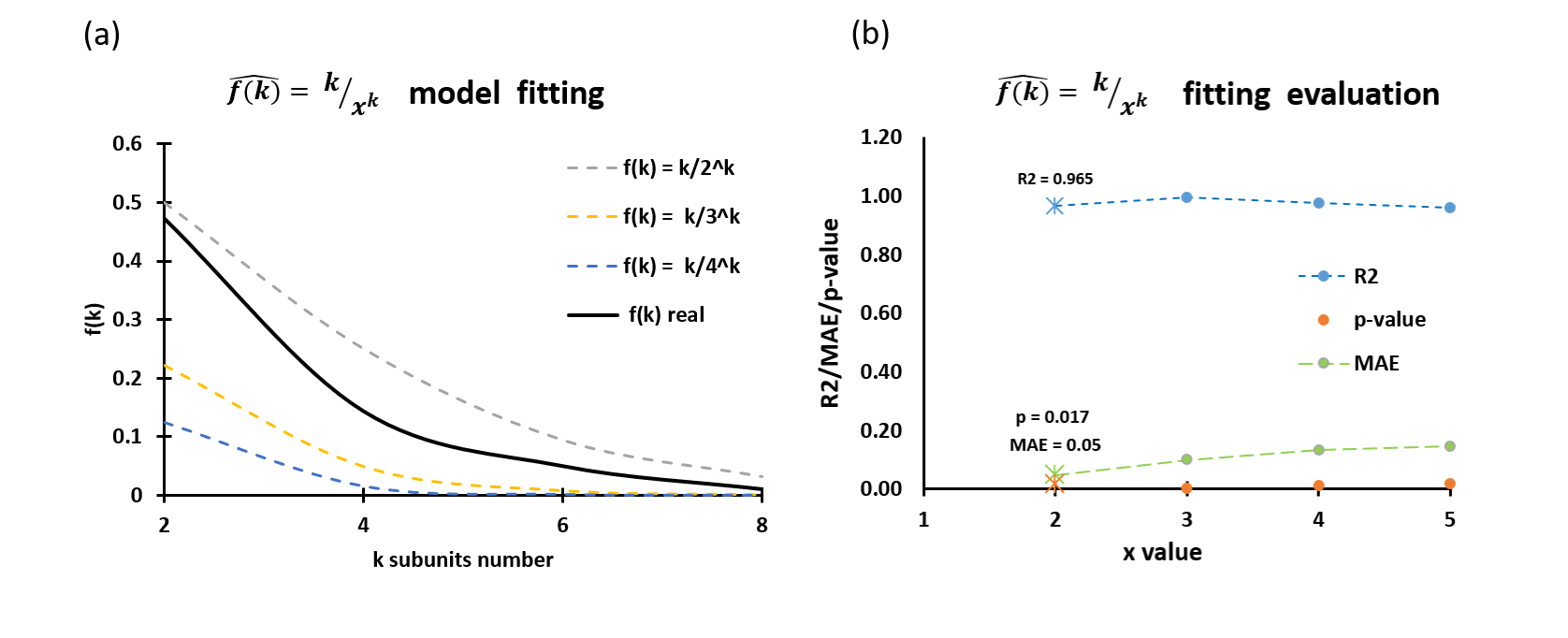


## Supplementary Figure S5: Fitting of protein even homo-oligomers distribution over biological pathways in yeast.

###
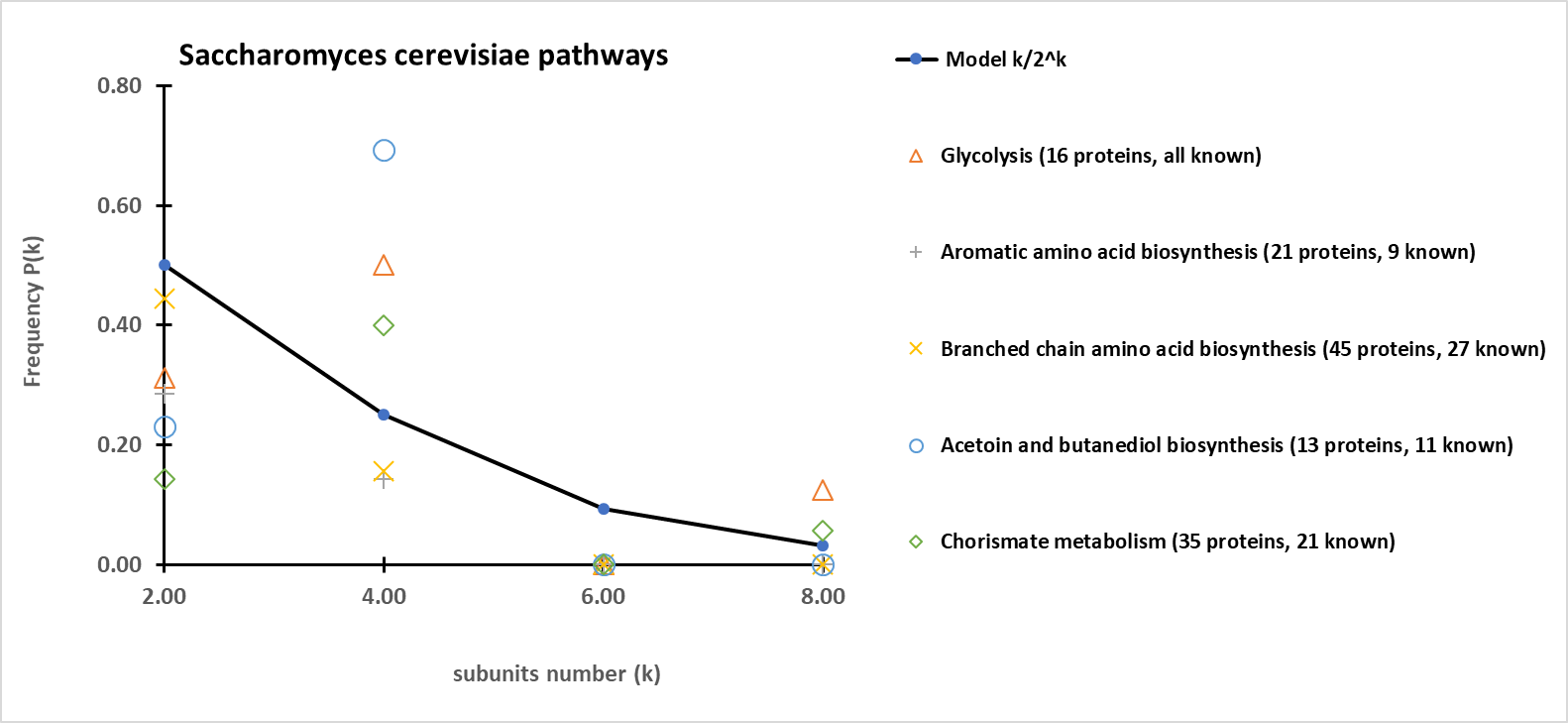
Protein even homo-oligomers distribution (*f_s_*=*N/N_T_*) over biological pathways by the resource-precision in Saccharomyces cerevisiae based on SGD database^5^. Several monomers, complexes and proteins with unknown oligomerization degree that are involved in these pathways were neglected. Following pathways was used for calculation: glycolysis, aromatic amino acid biosynthesis, branched chain amino acid biosynthesis, acetoin and butanediol biosynthesis, chorismate metabolism.

**Supplementary Figure S6: Protein** **abundance and homo-oligomers frequency**

Most proteins have a typical functionally effective abundance level. To answer the question if homo-oligomers frequency over proteome is expressed in organism protein abundance, we calculated normalized abundance level based on PaxDb^4^ database. In addition, PaxDb proteins data was mapped to UniProt^1^ repository by STRING^6^ protein ID to add subunits structure data. Then all proteins in organism dataset were grouped by subunits number *k*, where *k* = 1, 2…8. Total abundance was calculated for each group and normalized by total abundance per organism dataset for *E. coli, M. tuberculosis, B. Henselae, S. pombe, S. cerevisiae, R. norvegicus, M. musculus* and *H. sapiens.*

As shown in Fig. S6.a, most organisms have abundance pattern similar to homo-oligomers frequency over proteome. However, homodimers abundance is higher in *E. coli* (81%) *and M. tuberculosis* (66%) than typical frequency of homodimers (53%). In both species the homo-tetramers abundance is lower in *E. coli* (6%) and *M. tuberculosis* (9%) than the typical frequency of homo-tetramers (12%).

To test the hypothesis if resource precision model can also predict even protein homo-oligomers relative abundance in cell and explain the cell information capacity, we fit all organism abundance level of protein with even subunits number to *f(k)* = *k/2^k^* (Fig. S6.b).We found that model give significant fit (*R*^2^ > 0.9*, p* <0.05) for six from eight organisms. Following previous results, *E. coli* and *M. tuberculosis* abundance level are not fitted well. The determination coefficient *R*^2^ is above 0.8, but *p* >= 0.05.

(a) Absolute proteins abundance distribution in eight organisms, was calculated based on PaxDb data. The figure also contains data set size as defined by PaxDb and percentage of protein with known oligomeric structure after mapping with UniProt repository.

(b) Fitting proteins abundance with resource precision model *f(k)* = *k/2^k^*. The figure also contains the determination of fit quality *R*^2^ and fit statistical significance *p-*value.


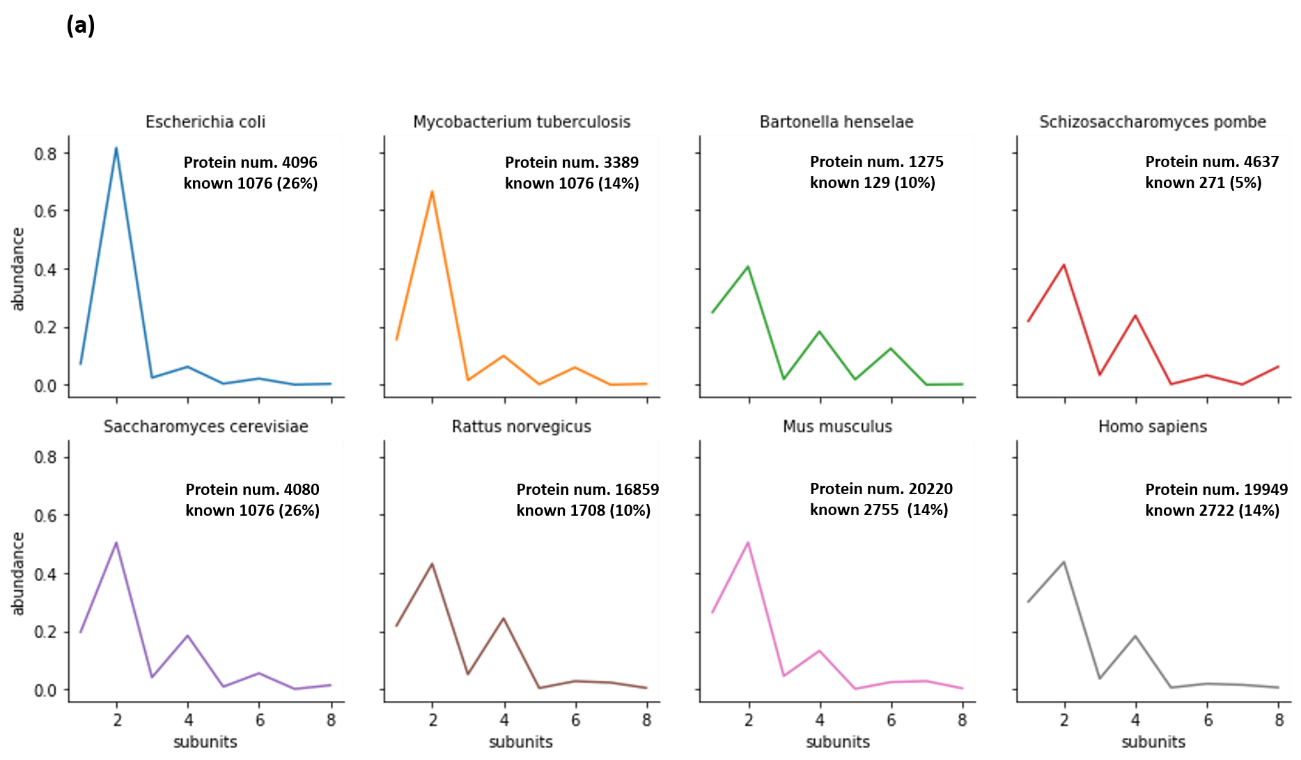


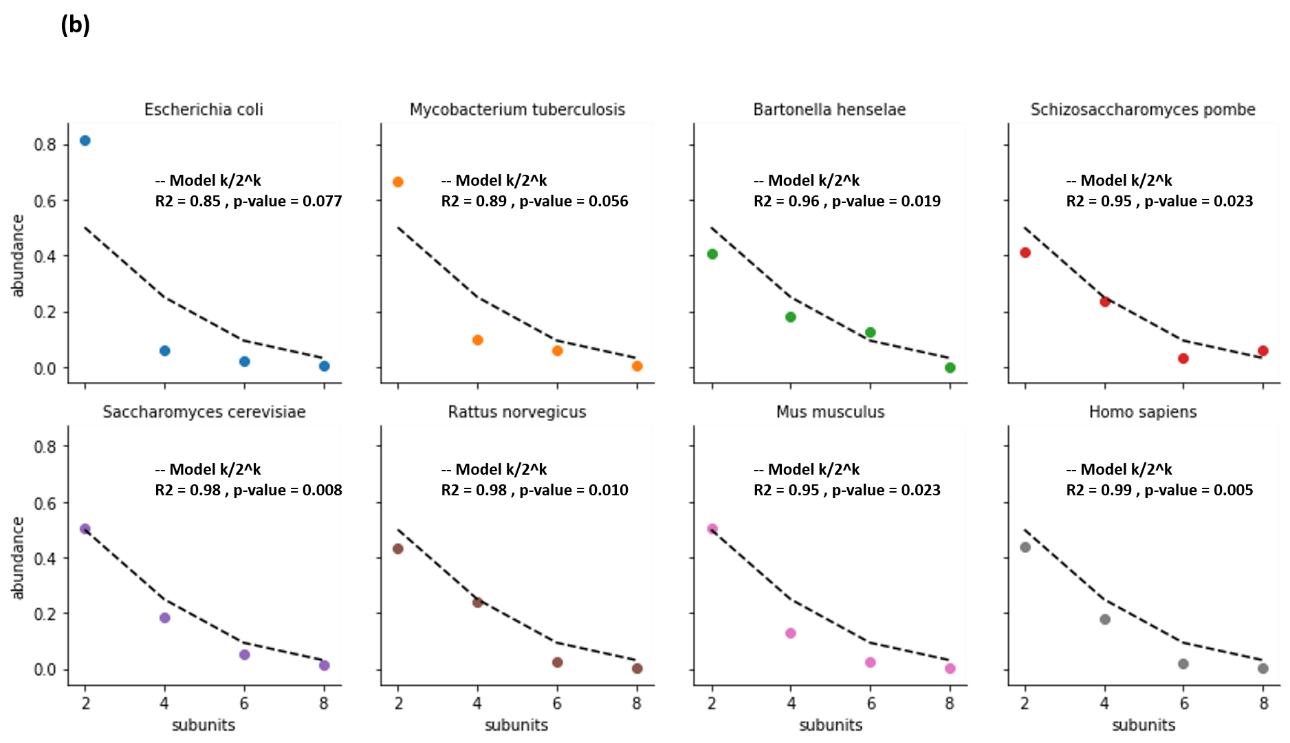


# Transparent Methods

## Python 3 software and all data file used for calculation can be found under:

<https://github.com/LenaDanielli/Protein-homo-oligomer-distribution>

## Supplementary Method S1: Protein-homo-oligomer distribution calculation

Protein_classification.ipynb python notebook is calculating frequency of proteins with different subunits number for nine organisms’ whole proteomes as listed in Table S2 and based on GO classification as defined in Table S3. To reproduce calculation, all repository including the data files need to be downloaded. Jupiter Notebook free access software needs to be installed to run the Protein_classification.ipynb file.

## Supplementary Method S2: Protein-homo-oligomer abundance distribution calculation

Protein_classification_abundance.ipynb python notebook calculates the abundance distribution of proteins with different subunits number for eight organisms as listed in Table S4 and based on mapping as defined in Table S5. To reproduce calculation, all repository including the data files needs to be downloaded. Jupiter Notebook free access software need to be installed to run the Protein_classification_abundance.ipynb file.

## References:

1. Bateman, A. UniProt: A worldwide hub of protein knowledge. Nucleic Acids Res. (2019) doi:10.1093/nar/gky1049.

2. Ashburner, M. et al. Gene ontology: Tool for the unification of biology. Nature Genetics (2000) doi:10.1038/75556.

3. Carbon, S. et al. The Gene Ontology Resource: 20 years and still GOing strong. Nucleic Acids Res. (2019) doi:10.1093/nar/gky1055.

4. Wang, M., Herrmann, C. J., Simonovic, M., Szklarczyk, D. & von Mering, C. Version 4.0 of PaxDb: Protein abundance data, integrated across model organisms, tissues, and cell-lines. Proteomics (2015) doi:10.1002/pmic.201400441.

5. Cherry, J. M. et al. Saccharomyces Genome Database: The genomics resource of budding yeast. Nucleic Acids Res. (2012) doi:10.1093/nar/gkr1029.

6. Szklarczyk, D. et al. STRING v11: Protein-protein association networks with increased coverage, supporting functional discovery in genome-wide experimental datasets. Nucleic Acids Res. (2019) doi:10.1093/nar/gky1131.
